# MAB21L1 modulates gene expression and DNA metabolic processes in the lens placode

**DOI:** 10.1242/dmm.049251

**Published:** 2021-12-23

**Authors:** Ryuichi Yamada, Akira Oguri, Katsunori Fujiki, Katsuhiko Shirahige, Yoshikazu Hirate, Masami Kanai-Azuma, Hirotaka Takezoe, Yoshihiro Akimoto, Naoki Takahashi, Yoshiakira Kanai

**Affiliations:** 1 Department of Veterinary Anatomy, the University of Tokyo, Tokyo 113-8657, Japan; 2 Department of Applied Biological Chemistry, the University of Tokyo, Tokyo 113-8657, Japan; 3 RNA Company Limited, Tokyo 144-0051, Japan; 4 Laboratory of Genome Structure and Function, Institute for Quantitative Biosciences, the University of Tokyo, Tokyo 113-0032, Japan; 5 Department of Experimental Animal Model for Human Disease, Center for Experimental Animals, Tokyo Medical and Dental University, Tokyo 113-8510, Japan; 6 Genble Inc., Fukuoka 814-0001, Japan; 7 Department of Anatomy, Kyorin University School of Medicine, Tokyo 181-8611, Japan

**Keywords:** Mab21l1, Lens placode, scRNA-seq, Transmission electron microscopy

## Abstract

Mutations in human *MAB21L1* cause aberrations in lens ectoderm morphogenesis and lead to congenital cerebellar, ocular, craniofacial and genital (COFG) syndrome. Murine *Mab21l1*-null mutations cause severe cell-autonomous defects in lens formation, leading to microphthalmia; therefore, *Mab21l1*-null mice are used as a mouse model for COFG syndrome. In this study, we investigated the early-onset single-cell-level phenotypes of murine *Mab21l1*-null lens ectoderms using electron microscopy and single-cell RNA sequencing (scRNA-seq). Electron microscopy and immunohistochemical analyses indicated endoplasmic reticulum stress at the 24- to 26-somite stage in *Mab21l1*-null lens placodes. scRNA-seq analysis revealed that 131 genes were downregulated and 148 were upregulated in *Mab21l1*-null lens ectoderms relative to the wild type. We successfully identified 21 lens-specific genes that were downregulated in *Mab21l1*-null cells, including three key genes involved in lens formation: *Pitx3*, *Maf* and *Sfrp2*. Moreover, gene ontology analysis of the 279 differentially expressed genes indicated enrichment in housekeeping genes associated with DNA/nucleotide metabolism prior to cell death. These findings suggest that MAB21L1 acts as a nuclear factor that modulates not only lens-specific gene expression but also DNA/nucleotide metabolic processes during lens placode formation.

## Abstract (precis)

**Summary:** MAB21L1 acts as a nuclear factor that modulates not only lens-specific gene expression but also DNA/nucleotide metabolic processes during lens placode formation.

## INTRODUCTION

In mouse and human embryos, eye specification is initiated in the forebrain, after which the wall of the forebrain evaginates and comes into close contact with the head surface ectoderm. Lens morphogenesis begins with the thickening of the lens ectoderm, which covers the eye field to form the lens placode. The invagination and proliferation of lens placode cells give rise to lens vesicles, whereas the remaining surface ectoderm cells contribute to cornea and iris formation, becoming the anterior elements of the eye. In mice, lens progenitor cells are characterized by the expression of lens lineage-specific transcription factors, such as *Pax6*, *Six3*, *Sox2*, *Mab21l1* and *FoxE3* (Cvekl and Duncan, 2007). Disruption of any of the genes involved in lens development leads to severe eye abnormalities (Cvekl and Zhang, 2017). Owing to the failure of these mutants to initiate or complete lens placode formation, unraveling the molecular mechanisms and events that drive lens placode formation has been challenging.

The lens placode is completely absent or severely developmentally arrested in mouse embryos harboring null mutations in *Pax6* (Hill et al., 1992) or *Six3* (Liu et al., 2010). Moreover, ectopic expression of *Pax6* in frog (Chow et al., 1999) and *Six3* in fish (Oliver et al., 1996) can generate ectopic lenses, suggesting that these genes are sufficient to induce lens development. A variety of experimental approaches, ranging from tissue recombination studies to genetic loss of function, have identified *Pax6* as the key transcription factor for inducing the lens placode (Fujiwara et al., 1994; Ashery-Padan et al., 2000). As the regulatory interaction between *Six3*, *Pax6* and *Sox2* paves the way for the expression of the essential crystallin regulatory genes *Maf* and *Prox1*, decades of molecular studies have focused on deciphering the hierarchy of this tripartite network (Cvekl and Ashery-Padan, 2014; Cvekl and Zhang, 2017). Extensive studies using conditional deletion, chromatin immunoprecipitation, an electrophoretic mobility shift assay and luciferase reporter assays have provided the current model, whereby *Six3* directly activates *Pax6* and *Sox2* expression in the presumptive lens ectoderm (Liu et al., 2006).

In *FoxE3* and *Pitx3* mutant mice, the lens vesicle forms but fails to separate from the surface ectoderm, leading to disorganized rudimentary lenses (Ho et al., 2009). Notably, *Mab21l1-*null mice exhibit severe cell-autonomous defects in lens placode invagination due to impaired cell proliferation and survival (Yamada et al., 2003); this provided a unique model with which to study early lens placode formation.

The *mab-21* gene was first identified as a cell fate determination gene that regulates sensory ray morphogenesis in male nematodes (Baird et al., 1991). *Mab-21* is highly conserved in a wide range of invertebrates and vertebrates, and two *mab-21* orthologs, *Mab21-like 1* (*Mab21l1*) and *Mab21-like 2* (*Mab21l2*), have been identified in several species, including mice and humans (Mariani et al., 1999; Wong and Chow, 2002). In vertebrates, members of this gene family encode proteins that share more than 90% amino acid sequence homology (Mariani et al., 1999; Wong and Chow, 2002). Despite several conserved structural similarities between MAB21L1 and cyclic guanosine monophosphate (cGMP)-adenosine monophosphate (AMP) synthase (cGAS), the biochemical function of MAB21L1/2 remains elusive, although MAB21L1/2 exhibits a mild affinity of MAB21L1/2 for nucleic acids *in vitro* (de Oliveira Mann et al., 2016).

*Mab21l2*-deficient mice show severe developmental defects of the heart, liver, eye and ventral body wall, which can result in embryonic death (Saito et al., 2012; Yamada et al., 2004). In humans, *MAB21L2* mutations cause similar defects, with the addition of eye malformations and skeletal dysplasia (Rainger et al., 2014). *Mab21l1*-deficient mice can survive and grow to adult developmental stages (Yamada et al., 2003); however, they display severe microphthalmia and mild atrophy of the preputial glands with the same abdominal ectoderm origin as the human scrotum (Yamada et al., 2003). Similar to *Mab21l1*-deficient mouse phenotypes, human *MAB21L1* mutations cause various ocular abnormalities, such as microphthalmia, coloboma and/or cataracts (Seese et al., 2021), in addition to facial dysmorphisms, cerebellar hypoplasia and scrotal agenesis; this disease is called cerebellar, ocular, craniofacial and genital (COFG) syndrome (Rad et al., 2019). Zebrafish *mab21l1*-null mutants also exhibit aberrant morphogenesis of the lens and cornea, similar to mice and humans with *Mab21l1* mutations (Seese et al., 2021). Together, these studies suggest that *Mab21l1* plays a crucial conserved role in lens ectoderm morphogenesis in vertebrates. However, MAB21L1 deficiency causes rapid cell loss within the lens ectoderm area during the early organogenic embryo stage (Yamada et al., 2003; Seese et al., 2021). Owing to the rapid death of the majority of lens placode cells, the molecular and cellular events that occur immediately downstream of *Mab21l1* in the lens ectoderm are currently unknown.

Recent technical advances in single-cell RNA sequencing (scRNA-seq) have enabled the sequencing and quantification of transcriptomes from samples containing relatively low numbers of cells. In a previous study, we applied scRNA-seq to individual cells collected from the approximate eye area of mouse early somite-stage embryos and successfully identified key stage-dependent structural and metabolic genes involved in initial retinal specification (Yamada et al., 2021). In this study, we used scRNA-seq and electron microscopy to characterize the pathological phenotypes and transcriptome profiles of the lens ectoderm at the single-cell level immediately prior to apoptotic cell death in *Mab21l1*-null mutants. This revealed the possible functions of *Mab21l1* in the lens placode during early ocular morphogenesis.

## RESULTS

### MAB21L1 mainly localizes to the nuclei of lens placode cells

**Fig. 1.**
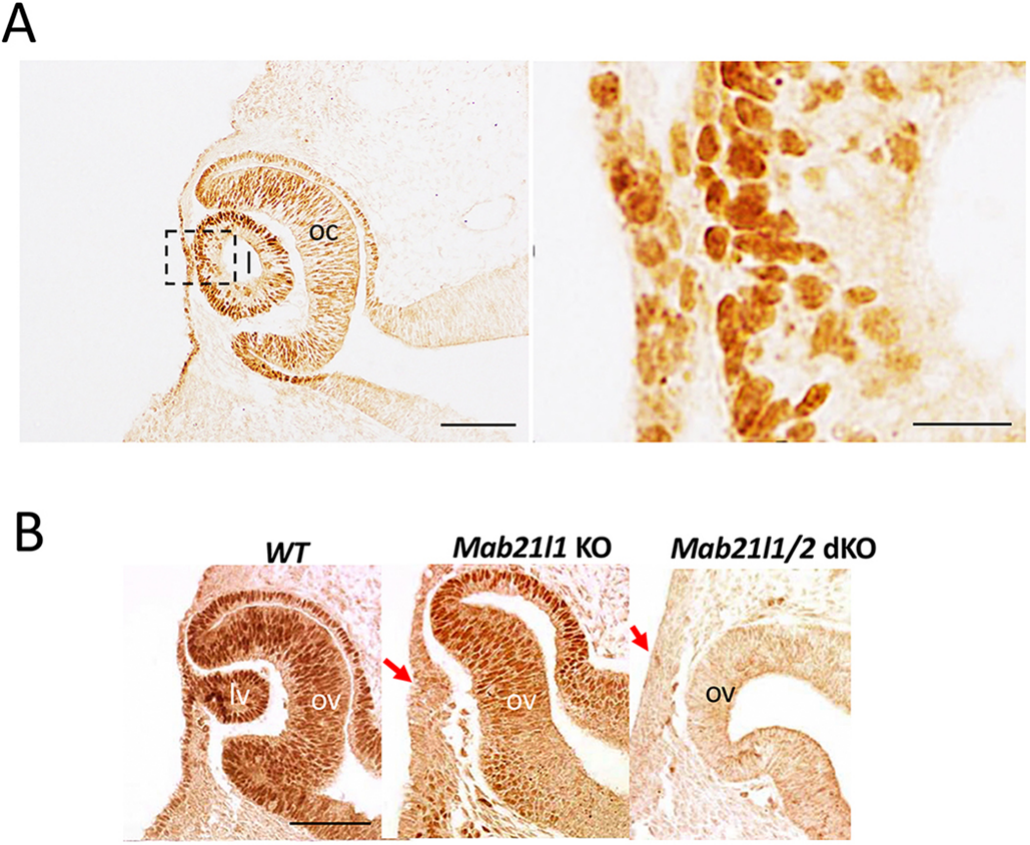
**Nuclear localization of the MAB21L1 protein in the developing lens.** (A) Anti-MAB21L1 immunostaining (brown). Intense positive signals were detected in the nucleus of the lens, as well as the surface ectoderm, optic cup and retinal pigmented epithelium at E11. The image on the right shows a higher magnification view of the lens and its surface ectoderm, indicated by a dashed box in the left panel. (B) Anti-MAB21L1 immunostaining of the lens and optic vesicle of wild-type (WT, left), *Mab21l*-single knockout (KO, middle) and *Mab21l1/Mab21l2*-double KO (right) embryos. No MAB21L1^+^ signals were present in the lens ectoderm of the *Mab21l1*-single KO or *Mab21l1/Mab21l2*-double KO (red arrows). Some crossreactive signals with MAB21L2 were present in the optic vesicles of *Mab21l1*-single KO embryos (middle panel). L, lens; lv, lens vesicle; oc, optic cup; ov, optic vesicle. Scale bars: 50 µm (A, left panel and B); 10 µm (A, right panel).

MAB21-domain proteins serve functions in both the nucleus and the cytoplasm (Deml et al., 2015; Mariani et al., 1999). Here, we investigated the subcellular localization of MAB21L1 in developing lens placodes *in vivo* at embryonic day (E)9.5. To examine endogenous MAB21L1 *in vivo*, we generated anti-MAB21L1 polyclonal antibodies and performed immunohistochemical analyses. MAB21L1^+^ signals were mainly present in the nuclei of lens placode, surrounding surface ectoderm (i.e. the developing cornea and iris) and optic vesicle cells (Fig. 1A). Moreover, nuclear MAB21L1 signals were not detected in the defected lens pits of single *Mab21l1-*null or double *Mab21l1/Mab21l2*-null embryos (Fig. 1B). These findings suggest that MAB21L1 may function as a lens placode-specific nuclear factor *in vivo*. In addition, MAB21L1 signals were detectable in the optic vesicles of wild-type and single *Mab21l1-*null embryos but not in those of *Mab21l1/Mab21l2* double-null embryos (Fig. 1A,B); this suggests that the anti-MAB21L1 antibodies cross-reacted with MAB21L2, which shares 94% amino acid sequence similarity with MAB21L1 (Mariani et al., 1999).

### *Mab21l1*-null lens placode cells have enlarged endoplasmic reticula immediately prior to cell death

**Fig. 2.**
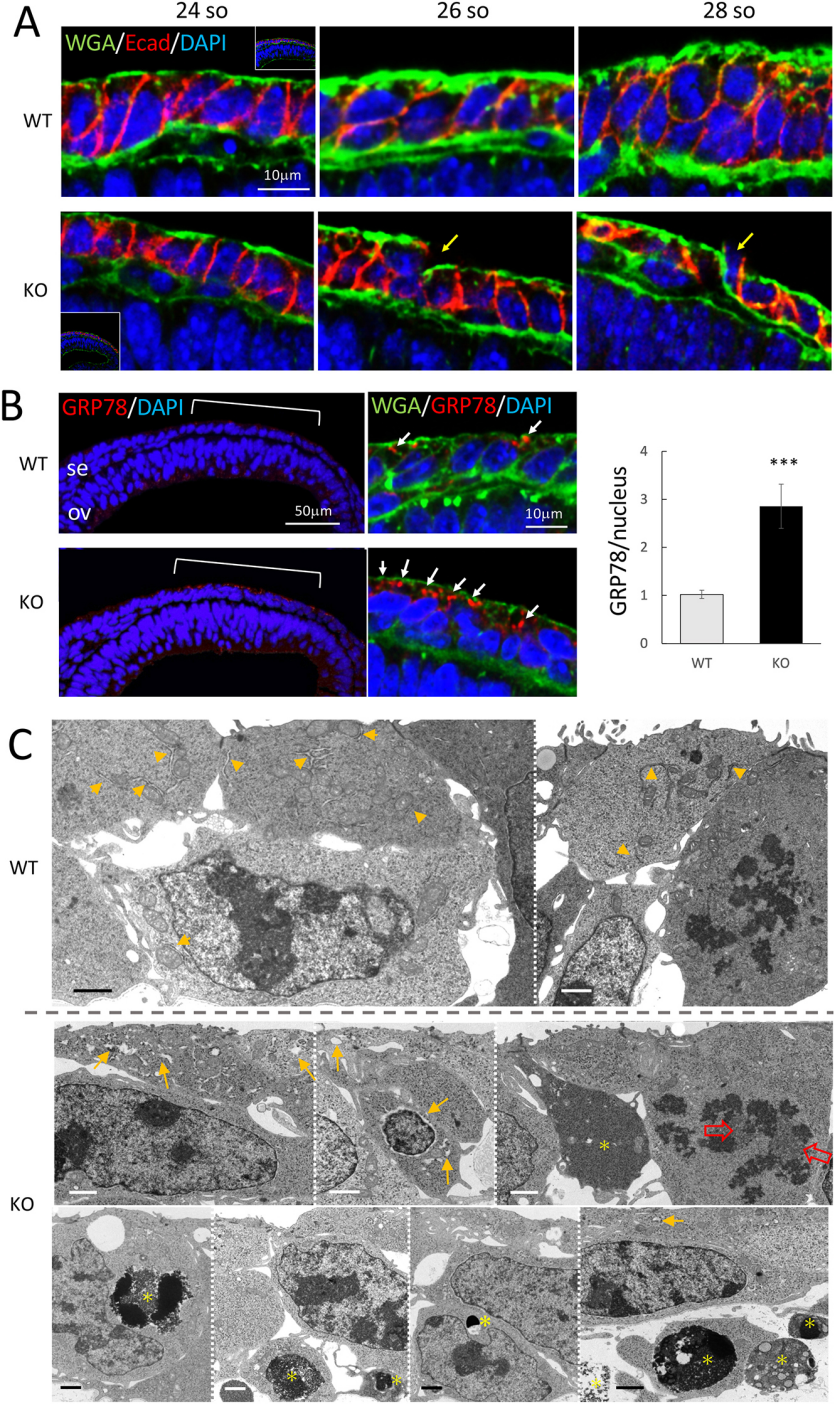
**Early-onset *Mab21L1*-KO phenotypes in the lens ectoderm cells prior to apoptotic cell death at the 24- to 26-somite stage.** (A) Lens ectoderm staining with WGA (green), anti-E-cadherin (Ecad; red) and DAPI (blue) of developing wild-type (WT) and *Mab21l1*-KO lens ectoderm layers at 24-, 26- and 28-somite stages. Discontinuous WGA^+^ glycocalyx layers are present in KO lens placodes, even at the 28-somite stage (yellow arrows). Insets in the 24-somite panels (left) contain a lower magnification view. (B) Staining of the wild-type and KO lens ectoderms at 24- to 25-somite stages with anti-78 kDa glucose-regulated protein (GRP78, red), WGA (green) and DAPI (blue). Several intense GRP78^+^ signals were present in the apical cytoplasm of the KO lens ectoderm (white arrows). Bar graph (right panel) shows the number of GRP78^+^ foci/number of nuclei (mean±s.e.m.). *n*=15. ***P<0.001 (unpaired two-tailed Student's *t*-test). ov, optic vesicle; Se, surface ectoderm. (C) Transmission electron microscopy of wild-type (upper panels) and KO (lower panels) lens ectoderms at the 24-26-somite stage (*n*=4). Several KO lens placode cells exhibited enlarged ER lumens (yellow arrows) and aberrant mitotic chromosome aggregation (open red arrows). Dead cells, cell debris and phagosomes (yellow asterisks) were present in the epithelial layer (bottom panels) and the lower mesenchymal space (bottom-right panel) of the KO lens area. Yellow arrowheads indicate normal ER in the wild-type lens ectoderm. Scale bars: 1 µm (C).

Signs of severe defects in lens placode growth, such as reduced bromodeoxyuridine (BrdU) uptake and increased apoptotic signals, are present in *Mab21l1*-null mouse embryos in the 30-somite stage during the onset of lens placode invagination (Yamada et al., 2003). To determine the first cellular defects and the timing of their onset in *Mab21l1-*null lens placodes, we performed immunohistochemistry on lens placodes during the 24- to 30-somite stage (E9.25-E9.75) using wheat germ agglutinin (WGA) to stain for apical surface glycocalyces and Golgi apparatuses, as well as stains for 78 kDa glucose-regulated protein (GRP78), a marker for rough endoplasmic reticula (ER) and ER stress (Xu et al., 2005), and E-cadherin (Ecad), a basolateral membrane marker (Fig. 2A,B).

At the 24-somite stage, the WGA and Ecad staining patterns of the lens placodes of *Mab21l1*-null and wild-type littermates were similar, with both types exhibiting typical single cuboidal epithelia with discontinuous WGA^+^ glycocalyx apical surface layers (Fig. 2A). At the 26-somite stage, the wild-type epithelial cells had elongated and the WGA^+^ signals along their apical surface had thickened, leading to placode formation by the 28-somite stage (upper panels in Fig. 2A). By contrast, *Mab21l1*-null lens placodes exhibited no significant changes in epithelial structure during the 24- to 28-somite stages, and discontinuous WGA^+^ signals were still found along the epithelial apical surface (lower panels in Fig. 2A). These results suggest that the earliest *Mab21l1*-null phenotype is defective epithelial cell maturation in the lens placode at the 24- to 26-somite stages.

Anti-GRP78 staining in wild-type and *Mab21l1*-null lens placodes were compared at the 24- to 26-somite stages. GRP78^+^ signals were present in the apical cytoplasm of the wild-type lens placode (Fig. 2B). Interestingly, intense GRP78 signals were present in the apical cytoplasm of *Mab21l1*-null lens placode cells (Fig. 2B). The number of GRP78^+^ foci per nuclei was significantly higher in *Mab21l1*-null compared to wild-type lens placodes (Fig. 2B), which suggests possible ER stress in the *Mab21l1*-null lens placode immediately prior to placode formation.

Ultrastructural transmission electron microscopy analysis revealed the presence of enlarged ER lumens in the apical cytoplasm of *Mab21l1*-null lens placode cells (Fig. 2C), corresponding to the GRP78^+^ signals observed in Fig. 2B. Moreover, in *Mab21l1*-null lens placodes, the mitotic chromosomes were aberrantly aggregated in some *Mab21l1*-null lens placode cells (Fig. 2C, open red arrows). Dead cells, cell debris and phagosomes were present inside the lens epithelia (Fig. 2C, yellow asterisks), in addition to apoptotic cell debris in the extracellular space between the lens placode and optic vesicle layers (Fig. 2C, bottom right; Fig. S1). No significant differences in the ultrastructural features of wild-type and *Mab21l1*-null apical surface membranes or cilia/microvilli were detected via transmission or scanning electron microscopy at the 24-26 somite stages (Figs S1, S2). Similar to the wild type, single primary cilia and prominent microvillus borders were present even at the 28-somite stage (Fig. S2, yellow arrows) at the apical surfaces of *Mab21l1*-null lens placodes. However, long cell protrusions on irregular apical surfaces were present in some *Mab21l1*-null lens placode cells (Fig. S2B, blue arrows), suggesting a possible repair response in resealing their damaged epithelial sheet (also see the gene ontology terms ‘bicellular tight junction assembly’ and ‘regulation of actin filament-based process’ in 148 genes upregulated in *Mab21l1*-null lens placodes, as described in the following scRNA-seq paragraphs and Fig. S3).

### scRNA-seq of the cluster of ectoderm cells covering the eye area of 26-somite stage wild-type and *Mab21l1*-null embryos

**Fig. 3.**
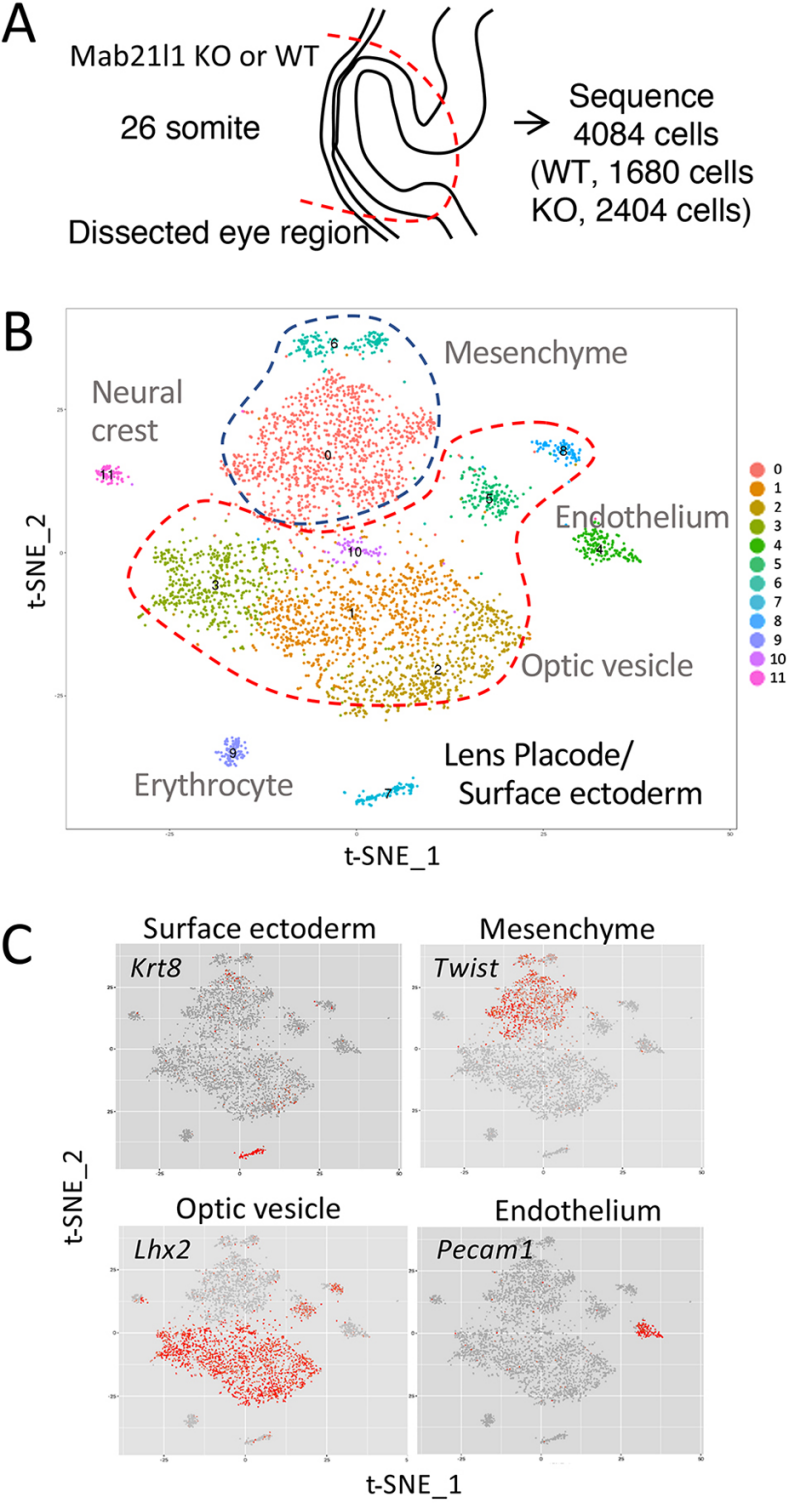
**scRNA-seq of the ocular area identified 12 major cell types, including a lens ectodermal cluster.** (A) A schematic image of a 26-somite stage embryo. The red dashed line indicates the dissected eye region subjected to scRNA-seq. KO, knockout; WT, wild-type. (B) t-SNE plot of all cells that passed quality control (*n*=4084 cells) colored according to cluster type. (C) t-SNE feature plots showing expression of the surface ectoderm (*Krt8*), optic vesicle (*Lhx2*), mesenchymal (Twist) and endothelium (*Pecam1*) marker genes. Cellular expression is colored on a scale from low level (gray) to high level (red).

To identify the initial molecular defects in the ectodermal area of the lens placode of *Mab21l1*-null embryos, we performed scRNA-seq using cells from the approximate eye area of embryos at the 26-somite stage using a 10x genomics chromium system (Fig. 3A). A total of 4084 individual cells were analyzed (1680 and 2404 cells from wild-type and *Mab21l1*-null embryos, respectively), from which the cells could be categorized into seven major clusters (Fig. 3B). As in our recent scRNA-seq study (Yamada et al., 2021), the expression of annotated marker genes was used to identify each cluster, using a combination of the Mouse Atlas, LifeMap Discovery and Mouse Genome Informatics databases. We identified one cluster that had high ectoderm marker expression levels (cluster 7: *Krt8*, *Krt18*, *Dlx5* and *Dlx6*), six clusters with high optic vesicle marker expression (clusters 1, 2, 3, 5, 8 and 10: *Lhx2* and *Rax*) and two clusters with high mesenchymal marker expression (clusters 0 and 6: *Col3a1* and *Prrx2*). Other clusters were associated with the endothelium (cluster 4: *Pecam1* and *Cd34*), erythrocytes (cluster 9: *Hbb-bs*, *Hbb-bt*, *Alas2* and *Gypa*), neural crests (cluster 11: *Sox10*, *Zeb2* and *Ednrb*) and lymphocytes (cluster 12: *Cd52*, *Fcer1g* and *C1qb*) (Fig. 3B,C). The differentially expressed genes (DEGs) for each cluster are listed in Table S1.

### Transcriptional changes immediately downstream of *Mab21l1* in lens placodes

**Fig. 4.**
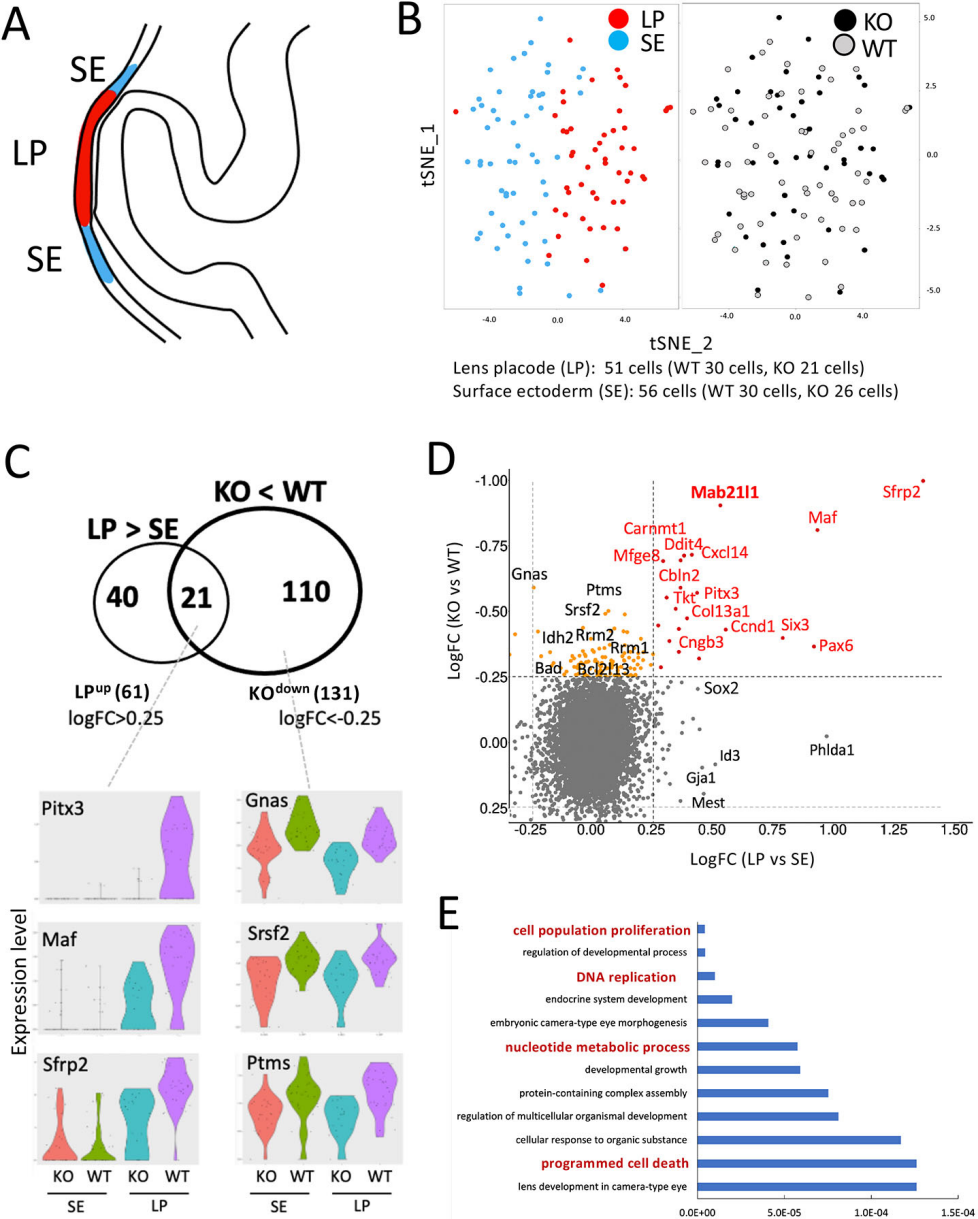
**Twenty-one lens placode genes were downregulated in *Mab21l1*-KO lens ectoderms.** (A) A schematic lens ectoderm image showing the distribution of two cell types: lens placode (LP, red) and surface ectoderm around lens placode (SE, blue). (B) t-SNE plots showing LP (red) and SE (blue) cells extracted from cluster 7 (see Fig. 3B). Data points represent cells, which are colored according to cluster type (LP versus SE, left) and genotype (KO versus WT, right). Cell numbers are indicated below the plots. (C) Venn diagram showing the 61 genes upregulated in lens placode cluster cells compared to surface ectoderm cluster cells (LP^up^), and the 131 genes downregulated in the KO cluster compared to the wild-type cluster; 21 LP^up^ genes were also downregulated in KO (KO-LP^down^). Violin plots showing the expression levels of *Pitx3*, *Cbln2*, *Cngb3*, *Gnas*, *Srsf2* and *Ptms* are shown as representative genes downregulated in KO compared to wild type. (D) Scatter plot showing the logFC value of each gene for KO versus WT in the *y*-axis, and for LP versus SE in the *x*-axis. Red dots represent the 21 KO-LP^down^ genes shared by the LP and KO groups, as shown in the Venn diagram in C. Orange dots represent the 110 genes downregulated in KO cells, which are not upregulated in the lens placode. The names of representative genes are indicated close to the dot. (E) The top 12 GO terms associated with the 131 genes downregulated in KO; lens/eye-related GO terms were omitted. GO terms mentioned in the Results and Discussion sections are highlighted in bold red font.

Next, we extracted and reanalyzed the scRNA-seq data for cluster 7, which contained lens ectoderm progenitor cells, and plotted the subclusters using T-distributed stochastic neighbor embedding (t-SNE), revealing two separate subclusters. Pax6, a lens marker gene, was upregulated in one subcluster, which we named lens placode (LP); the other subcluster, which we named surface ectoderm (SE), represented the remaining surface ectoderm cells surrounding the lens placode region (Fig. 4A,B). We obtained a similar number of lens placode and surface ectoderm cells from wild-type (WT) and *Mab21l1*-knockout (KO, null) embryos (Fig. 4B, right panel), suggesting that *Mab21l1* deficiency does not severely affect lens placode cell number at the 26-somite stage (Fig. 4B).

To identify genes involved in lens placode formation, we analyzed the 61 genes upregulated in lens placode relative to surface ectoderm (LP^up^; LogFC >0.25). LP^up^ represents genes that are highly expressed in the lens placode, including known lens-related genes, such as *Pax6*, *Six3*, *Sox2* and *Mab21l1*, as well as novel genes, such as *Phlda1* and *Id3*, which have not previously been associated with lens development (Table S2). These lists present unbiased gene expression data for both the lens placode and surface ectoderm regions. To identify genes associated with *Mab21l1*, we examined the 131 genes downregulated in *Mab21l1*-null cells relative to wild-type cells within the same cluster (KO^down^; LogFC <−0.25) (Table S3). To identify genes downregulated in *Mab21l1*-null cells in the lens placode subcluster, we compared KO^down^ and LP^up^ DEGs. Twenty-one genes, including *Mab21l1*, were shared by the LP^up^ and KO^down^ DEGs (KO-LP^down^; Fig. 4C,D; Table S4). Consistent with previous studies, several regulators of lens formation, including *Pitx3* (Ho et al., 2009), *Maf* (Kawauchi et al., 1999) and *Sfrp2* (Sugiyama et al., 2013), were present within this group, suggesting that these genes are likely to represent *Mab21l1* downstream targets involved in lens placode formation. Of the LP^up^ genes, 34% were downregulated in *Mab21l1*-null cells (21/61 genes), suggesting that tissue-specific gene expression was disrupted in the developing lens placodes of *Mab21l1*-null embryos. *Pitx3*, *Maf* and *Sfrp2* were downregulated in *Mab21l1*-null lens placode cells compared to wild-type lens placode cells (Fig. 4C). Furthermore, *Gnas*, *Srsf2* and *Ptms* were downregulated in both lens placode and surface ectoderm cells in *Mab21l1*-null embryos relative to wild-type embryos (Fig. 4C).

Enriched gene ontology (GO) terms in the 131 KO^down^ genes included ‘DNA replication’ (e.g. *Mcm5*, *Mcm7*, and *Pola2*; *P*=9.9E-06), ‘nucleotide metabolic processes’ (e.g. *Rrm1*, *Rrm2* and *Rnaseh2b*; *P*=5.8E-05) and ‘programmed cell death’ (e.g. *Ddit4*, *Bad* and *Pdcd4*; *P*=1.3E-04) (Fig. 4E). By contrast, we identified 148 DEGs that were upregulated in *Mab21l1*-null cells compared to wild-type cells (LogFC <−0.25), as well as 22 surface ectoderm-related genes, such as *Fermt1* and *Limch1* in KO-LP cells (24% surface ectoderm genes genes; 22/91 genes; Fig. S3A,B; Table S3). Interestingly, GO analysis of 148 KO^up^ genes yielded terms similar to the KO^down^ terms, such as ‘cell division’ (e.g. *Cenpe*, *Reep4* and *Rala*; *P*=5.6E-06), ‘mitotic nuclear division’ (e.g. *Ube2c*, *Knstrn* and *Tpx2*; *P*=1.2E-05), ‘mitotic spindle organization’ (e.g. *Spc25*, *Prc1* and *Plk2*; *P*=3.6E-05) and ‘regulation of mitotic cell cycle’ (e.g. *Pole*, *Cenpf* and *Ccnf*; *P*=2.3E-04), as well as ‘regulation of apoptotic process’ (e.g. *Cldn7*, *Epcam* and *Pak2*; *P*=3.8E-05) (Fig. S3C). These results suggest that, at this early-onset stage, *Mab21l1* deficiency may affect cell division processes via the reduced expression of housekeeping genes related to DNA replication/nucleotide metabolism in lens placode cells, together with a partial lens placode to surface ectoderm conversion in *Mab21l1*-null lens ectoderm cells. All DEGs and their relative expression levels (LogFC [KO versus WT] versus LogFC [LP versus SE]) are listed in Fig. S4.

### *Cbln2* and *Cngb3* are downregulated in the lens placodes of *Mab21l1-*null embryos and can act as novel markers

**Fig. 5.**
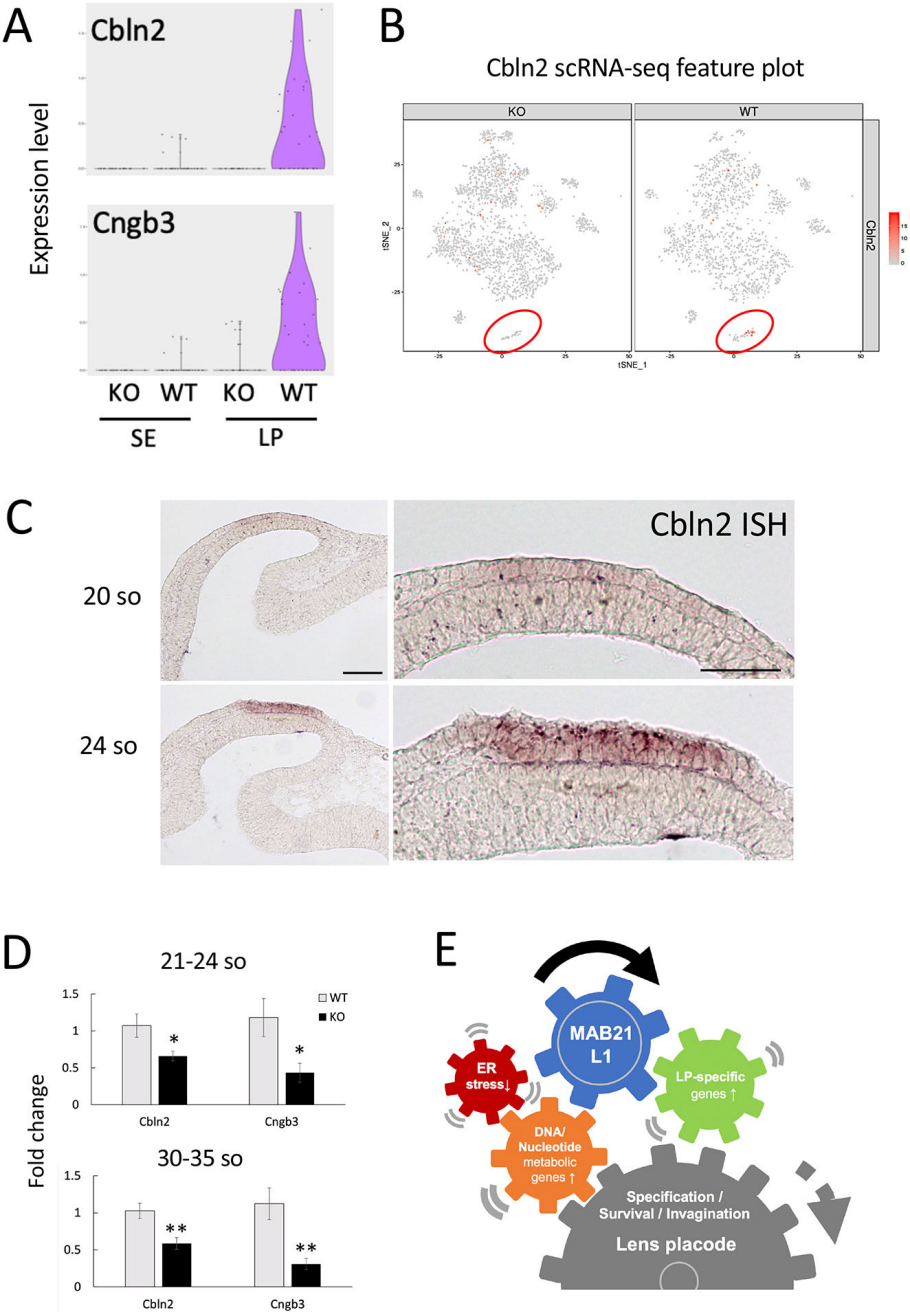
**Characterization of two genes, *Cbln2* and *Cngb3*, that are downregulated in *Mab21l1*-KO lens placodes.** (A) Violin plots showing the expression of *Cbln2* and *Cngb3*; expression is reduced in KO lens placodes. (B) t-SNE feature plots showing *Cbln2* expression in KO and wild-type (WT) cells. Red circles indicate cluster 7 cells (surface ectoderm/lens placode). (C) ISH analysis of *Cbln2* at the 20-somite (so; top) and 24-somite (bottom) stages. Scale bars: 100 µm (left panels); 50 µm (right panels). (D) Bar plots showing fold changes in *Cbln2* and *Cngb3* expression measured using qPCR (mean±s.e.m.). Top: pooled 21- to 24-somite stage samples. *n*=8. Bottom: pooled 30- to 35-somite stage samples. *n*=6. *P<0.05, **P<0.01 (unpaired two-tailed Student's *t*-tests). (E) Schematic representation of the molecular and cellular events immediately downstream of MAB21L1 during lens placode formation. MAB21L1 is a nuclear regulatory factor that upregulates several lens placode (LP)-specific genes, such as *Pitx3*, *Maf* and *Sfrp2*, leading to lens placode specification within the surface ectoderm covering the eye field. At the same time, MAB21L1 directly or indirectly regulates the expression of several housekeeping genes associated with DNA replication and nucleotide metabolic processes. In the lens ectoderm, MAB21L1 deficiency may cause nuclear DNA metabolic defects, as well as cytoplasmic ER stress, resulting in apoptotic cell death prior to lens invagination.

Among the 21 KO-LP^down^ genes, *Cbln2* and *Cngb3* were identified as novel lens placode genes that were downregulated in *Mab21l1*-null lens placode cells (Fig. 5A). Consistent with the scRNA-seq data (Fig. 5B), *in situ* hybridization (ISH) analysis revealed that *Cbln2* expression was initiated in the developing lens placode area at the 20-somite stage, and *Cbln2* expression levels increased at the 26-somite stage, specifically in the lens placode region (Fig. 5C). Then we examined the expression levels of *Cbln2* and *Cngb3* in whole-eye samples using qPCR. *Cbln2* and *Cngb3* expression levels were significantly lower in *Mab21l1*-null compared to wild-type whole-eye samples at both the 21-24-somite and 30-35-somite stages (Fig. 5D). By contrast, several of the 21 KO-LP^down^ genes, such as *Gnas*, *Srsf2* and *Ptms*, were ubiquitously expressed (Fig. S5). These results suggest that *Mab21l1* deficiency not only caused the downregulation of lens placode-specific genes but also altered the expression levels of ubiquitously expressed genes.

## DISCUSSION

*Mab21l1*-null mutations cause cell-autonomous defects in the growth and survival of lens placodes, which undergo apoptotic cell death and exhibit reduced BrdU uptake, and no lens placode invagination at the 30-somite developmental stage (Yamada et al., 2003). Owing to rapid cell loss within the small lens ectodermal area (∼200 cells in total), the molecular and cellular events driving the cell-autonomous defects in *Mab21l1*-null lens placodes prior to apoptotic cell death have, until now, been unclear. In this study, electron microscopy and immunohistochemical analyses of *Mab21l1*-null lens placodes revealed that the lens ectoderm defects in *Mab21l1*-null embryos commence at the 24- to 26-somite stage (E9.5), during which GRP78^+^ enlarged ER (i.e. ER stress; Yu et al., 1999; Xu et al., 2005) were present in lens placode cells prior to apoptosis. scRNA-seq of isolated *Mab21l1*-null lens ectoderm cells collected at the 26-somite stage revealed 131 downregulated and 148 upregulated DEGs in the lens ectoderm cells of *Mab21l1*-null embryos. GO terms, such as ‘programmed cell death’ (e.g. *Ddit4*, *Bad* and *Pdcd4*; *P*=1.3E-04) and ‘regulation of apoptotic process’ (e.g. *Cldn7*, *Epcam* and *Pak2*; *P*=3.8E-05), were associated with downregulated and upregulated DEGS, respectively. Furthermore, dead cells, cell debris and phagosomes were present within the lens epithelia and adjacent mesenchymal extracellular spaces (Fig. 2C). Together, the microscopy and scRNA-seq data revealed the initial transcriptional and physiological changes immediately downstream of MAB21L1 in lens ectoderm progenitors.

With the exception of ‘programmed cell death’ the GO terms associated with upregulated and downregulated genes in *Mab21l1*-null lens placode cells were similar; for example, ‘DNA replication’ (e.g. *Mcm5*, *Mcm7* and *Pola2*) and ‘nucleotide metabolic processes’ (e.g. *Rrm1*, *Rrm2* and *Rnaseh2b*) were associated with downregulated genes, whereas ‘cell division’, ‘mitotic nuclear division’, ‘mitotic spindle organization’ and ‘regulation of mitotic cell cycle’ were enriched in upregulated genes. This suggests that the expression of housekeeping genes associated with genome DNA metabolism in *Mab21l1*-null ectodermal cells is dysregulated. MAB21L1 has an N-terminal lobe with a subdomain that is structurally similar to the nucleotidyl transferase domain of cGMP-AMP synthase (de Oliveira Mann et al., 2016), which is involved in the recognition of cytosolic nucleic acids and the production of 2′,3′-cGMP-AMP (Ablasser et al., 2013; Gao et al., 2013). Notably, MAB21L1 can bind (albeit with weak affinity) to cytosine triphosphate nucleotides at a ligand–binding pocket at Arg62, which is outside the nucleotidyl transferase domain (de Oliveira Mann et al., 2016); this amino acid site is crucial for the biological function of MAB21L1 in zebrafish, as revealed by an *in vivo* complementation assay in the *mab21L2* loss-of-function zebrafish line (Seese et al., 2021). These data suggest that, in *Mab21l1*-null cells, the downregulation of DNA/nucleotide metabolic genes may be associated with the cytosine triphosphate binding function of MAB21L1.

The *Mab12l1* gene was initially identified as a target of homeobox C4 (HOXC4) (Tomotsune et al., 1993); a DNA fragment located ∼2 kb upstream of the putative *Mab21l1* transcription start site was found to bind to the HOXC4 protein in native chromatin (Yamada et al., 2003). MAB21L1 is mainly localized in the nuclei of lens ectoderm cells, suggesting the possible involvement of MAB21L1 in the expression of lens-specific genes in head surface ectoderm progenitors. The present scRNA-seq analysis revealed 21 downregulated lens placode genes and 22 upregulated surface ectoderm genes in lens placode cells of *Mab21l1*-null embryos, which indicates the defective specification of the lens ectoderm. Interestingly, *Six3* and *Pax6* were downregulated in *Mab21l1-null* lens placode cells compared to wild-type lens placode cells (Fig. 4D). These two key transcription factors, together with *Sox2*, regulate each other during lens placode formation (Cvekl and Ashery-Padan, 2014; Liu et al., 2006). As *Sox2* expression slightly appears decreased in KO-LP cells (Fig. 4D; also see Table S3), it is possible that MAB21L1 can directly regulate one and/or all of the expression of these genes specifically. However, we could not exclude the possibility that the nucleotide metabolic defects in *Mab21l1-null* lens placode may result in reduced levels of *Six3*, *Pax6* and *Sox2* transcripts.

Notably, three of the 21 genes, *Pitx3*, *Maf* and *Sfrp2*, were critical for lens development. *Pitx3* and *Maf* are required for lens formation (Kawauchi et al., 1999; Ho et al., 2009), and *Sfrp2*, which encodes a putative Wnt-Fz antagonist, is involved in lens epithelial cell development (Sugiyama et al., 2013). Therefore, the reduced expression of these three genes may have contributed to the defective lens placode formation phenotypes in *Mab21l1*-null embryos. Moreover, we found two novel marker genes, *Cbln2* and *Cngb3*, which are differentially regulated in MAB21L1-deficient lens placode cells. *Cbln2* is a synaptic organizer that contributes to brain function (Seigneur and Südhof, 2018). *Cngb3* is a cone photoreceptor with a cGMP-gated channel, which is associated with achromatopsia (Kohl et al., 2000). These findings suggest that *Cbln2* and *Cngb3* may be modulated by MAB21L1 during lens ectoderm specification; however, embryos with either *Cbln2-* or *Cngb3-*null mutations have not been shown to have defective phenotypes in their early lens morphogenesis (Seigneur and Südhof, 2018; Kohl et al., 2000).

In this study, we combined electron microscopy and scRNA-seq analyses, which enabled us to detect very early-onset single cell-level phenotypes at both the molecular and cellular level. We found that defects in nucleotide metabolism and ER stress may result in the loss of most lens placode cells prior to placode invagination. The dysregulated expression of genes such as *Gnas*, *Srsf2* and *Ptms* was not only detected in the lens placode but also in the surrounding surface ectoderm cells (Fig. 4C). We also found that several genes, such as *Cldn3* and *Hbb-y*, were upregulated in both the lens placode and surface ectoderm subpopulations (Fig. S3B), which suggests that similar defects were present throughout the head surface ectoderm area. The remaining surface ectoderm cells subsequently develop into the cornea and iris, other anterior elements of the eye. In human COFG syndrome, *MAB21L1* mutations cause a broad spectrum of ocular defects, such as lens-related microphthalmia/cataracts, corneal opacity/dystrophy and aniridia (Seese et al., 2021). In wild-type embryos, MAB21L1 is still expressed in the remaining surface ectoderm at later stages (see Fig. 1A,B), albeit at higher expression levels in lens placode compared to surface ectroderm subpopulations (Table S2), suggesting the continued function of MAB21l1 during the later stages of cornea and iris development. Our data indicate that the initial specification of surface ectoderm and lens placode cells is affected by MAB21l deficiency. Early-onset defects in surface ectoderm populations may contribute to the multiple ocular pathogeneses in human COFG syndrome (de Oliveira Mann et al., 2016; Rad et al., 2019; Seese et al., 2021).

Our proposed model for the function of MAB21L1 in the lens ectoderm is shown in Fig. 5E. Briefly, MAB21L1 likely acts as a nuclear factor that upregulates several lens placode-specific genes, including *Pitx3*, *Maf* and *Sfrp2*, leading to lens placode specification within the surface ectoderm layer covering the eye field. At the same time, MAB21L1 may modulate the expression of several housekeeping genes associated with DNA replication and nucleotide metabolic processes. Therefore, MAB21L1 deficiency may cause DNA metabolic defects, together with cytoplasmic ER stress, resulting in rapid cell death within the lens placode. Further studies examining the link between the nuclear localization of MAB21L1 and DNA replication/nucleotide metabolism are needed to elucidate the roles of MAB21L1 in the development of the lens placode and other organs, and to provide insight into the initial stages of COFG syndrome.

## MATERIALS AND METHODS

### Mice

*Mab21l1*- and *Mab21l2*-null mice (C57BL/6 strain) were generated as described previously (Yamada et al., 2003, 2004). *Mab21l/Mab21l2* double-null homozygous mice were obtained by crossing *Mab21l1/Mab21l2* double heterozygous males and females. All mouse care and experimental procedures were carried out in accordance with the Guidelines for Animal Experiments of the University of Tokyo and were approved by the Life Science Research Ethics and Safety group of the University of Tokyo, Japan (approval numbers P16–295, P18–046 and P18–145).

### Antibodies

Anti-MAB21L1 polyclonal antibodies were raised in a rabbit using His-MAB21L1 full-length recombinant protein as the antigen. Serum was collected and the antibodies affinity purified using GST-MAB21L1 full-length recombinant protein as bait. His-MAB21L1 was expressed in *Escherichia coli* BL21 cells and purified by extraction from a polyacrylamide gel. GST-MAB21L1 was expressed in *Spodoptera frugiperda* Sf9 cells using a Bac-to-Bac Baculovirus Expression System (Invitrogen, Waltham, MA, USA) and purified using glutathione sepharose 4B.

### Histology

Embryos at E9.0-E10.5 were dissected in PBS and fixed in 4% paraformaldehyde in PBS overnight at 4°C. For the frozen sections, embryos were fixed in 4% paraformaldehyde and PBS overnight at 4°C, then rinsed in PBS for 10 min. Next, the fixed embryos were cryoprotected in a sequential series of 10%, 20% and 30% sucrose dissolved in PBS. Then the embryos were placed in Tissue-Tek optimal cutting temperature compound (Sakura Finetek, Torrance, CA, USA) and rapidly frozen in liquid nitrogen. Sections (14 µm) were cut and mounted on MAS-coated glass slides (Matsunami Glass, Tokyo, Japan).

### Immunohistochemistry

Frozen sections were microwave-irradiated for 10 min in 10 mM citrate buffer (pH 6.0), incubated in 3% hydrogen peroxide for 10 min, and then incubated with primary antibodies overnight at 4°C. Anti-MAB21L1 rabbit polyclonal antibodies (generated in this study) were diluted to 1:100. Anti–E-cadherin mouse monoclonal antibodies (BD Transduction Laboratories, Franklin Lakes, NJ, USA, 610181) were diluted to 1:100. Anti-GRP78 rabbit polyclonal antibodies (Affinity BioReagents, Golden, CO, USA, PA1–014A) were diluted to 1:100. Antigen-antibody complexes were detected using a VECTASTAIN Elite ABC HRP kit (Vector Laboratories, Burlingame, CA, USA) and visualized with diaminobenzidine hydrochloride, goat anti-rabbit IgG (H+L) highly cross-adsorbed secondary antibody, Alexa Fluor Plus 594 (Invitrogen, A32740) 1:400, or donkey anti-Mouse IgG (H+L) highly cross-adsorbed secondary antibody, Alexa Fluor Plus 594 (Invitrogen, A32744). WGA-FITC (Vector Laboratories, FL-1021) was applied at a concentration of 5 µg/ml for 30 min at room temperature after secondary antibody incubation. Immunofluorescence images were obtained using a confocal microscope (Leica TCS SP8). As for GRP78 signal intensity, the number of GRP78^+^ foci/number of nuclei was counted in the lens placodes of wild-type and *Mab21l1*-null embryos at 24- to 26-somite stages, and then they were statistically analyzed by using unpaired Student's *t*-tests.

### Transmission and scanning electron microscopy

For transmission and scanning electron microscopy, the embryos at E9.25-E9.75 were fixed in 2.5% glutaraldehyde dissolved in 0.1 M phosphate buffer (pH 7.4) for at least 24 h at 4°C, and then postfixed using 1% osmium tetroxide (OsO_4_) dissolved in 0.1 M phosphate buffer (pH 7.3) for 1 h. For transmission electron microscopy, the embryos were postfixed with 0.5% OsO_4_ suspended in 0.1 M phosphate buffer (pH 7.3) for 30 min and dehydrated in a graded series of ethanol concentrations. After passage through propylene oxide, the tissues were embedded in Epon 812. Ultrathin sections were cut, stained with uranyl acetate and lead citrate, and then observed with a transmission electron microscope (JEOL, Tokyo, Japan, JEM-1010C). For scanning electron microscopy, the specimens were dehydrated in a graded series of ethanols (50%, 70%, 90%, 99.5%, and 100%), critical-point dried with carbon dioxide using a freeze-drying device (JEOL JFD-300), mounted and then coated with gold using a sputter coater. Finally, the specimens were observed under a scanning electron microscope (JEOL JSM-5600 LV SEM).

### *In situ* hybridization and qPCR

ISH was performed as described previously (Yamada et al., 2003). Briefly, frozen sections were hybridized *in situ* at 65°C in 50% formamide, 20 mM Tris-hydrochloric acid (pH 8.0), 300 mM sodium chloride, 0.2% Sarkosyl, 1× Denhart's solution, 10% dextran sulfate and 0.5 mg/ml yeast tRNA. *Cbln2* probes were prepared from commercially available full-length cDNA clones (Dharmacon, Lafayette, CO, USA). The probes were labeled with digoxigenin using standard procedures. For the expression levels of *Cbln2* and *Cngb3*, whole-eye cDNA samples were prepared from wild-type and *Mab21l1*-null embryos at both 21-24-somite and 30-35-somite stages, and were then analyzed by qPCR, as described previously (Nguyen et al., 2017). Briefly, total RNA was extracted using TRIzol reagent (Invitrogen), according to the manufacturer's instructions. cDNA was synthesized from 500 ng of total RNA using 5XPrimeScript RT Master Mix (TaKaRa Bio, Japan) according to the manufacturer's instructions. The expression levels of genes were quantitatively measured by real-time PCR using the following cycling parameters: denaturation for 30 s, followed by 40 cycles of denaturation at 95°C for 10 s, and annealing-extension at 60°C for 30 s. Reactions were performed in 10 μl volumes with 5 μl of SYBR Premix Ex Taq II (TaKaRa Bio), 4 μl of diluted cDNA, as described above, and 1 μl of 2 μM primers (final concentration 200 nM). Primer sets used in this study showed a single peak by post-amplification melting curve analysis. Transcription levels were normalized to ribosomal protein large P0 (*Rplp0*) expression. Primers for *Cbln2* and *Cngb3* were designed in this study using the Primer-BLAST tool available on the National Center for Biotechnology Information website, and were as follows: Cbln2_F, 5′-TGAGCAACCGTACCATGACC-3′; Cbln2_R, 5′-GGAGGCAAGGTCAAAGTGGT-3′; Cngb3_F, 5′-GAGTTCGGACTTGGCTGGAA-3′; and Cngb3_R, 5′-CATTGCTGTCGGGAGGTTCT-3′.

### Generation of the scRNA-seq library

The scRNA-seq library was generated using a 10x chromium system. A single embryo was dissected under a stereomicroscope, and a pair of optic areas were manually dissected using a fine needle (Austerlitz insect pins, 0.1 mm diameter). The dissected tissues were incubated in 0.05% trypsin-EDTA at 37°C for 3 min. Then, 10% fetal calf serum/minimal essential medium was added to stop the proteinase reaction. Next, the cells were dissociated by gentle pipetting, and suspended in 0.04% bovine serum albumin/PBS solution. The number of cells was determined using a Countess II FL automated cell counter (Invitrogen, Carlsbad, CA, USA), and 2000 cells/sample were prepared to generate the scRNA-seq library using a Chromium Single Cell 3′ Reagent Kit v3 (10x Genomics, Pleasanton, CA, USA). The Cell Ranger Single-Cell Software Suite was used to perform sample demultiplexing, barcode processing and single-cell 3′ gene counting. The cDNA insert was aligned to the mm10/GRCm38 reference genome.

The resulting data were derived from 4084 cells. Data from wild-type and *Mab12l1*-null samples were normalized and then integrated into a single dataset (gene-barcode matrix) using the Cell Ranger aggr program. Then the data were preprocessed using the R package Seurat (Stuart et al., 2019). Genes detected in fewer than three cells were eliminated from the analysis. We removed cells that expressed <1500 genes or >8000 genes, and cells with >0.05% of their transcripts containing mitochondrial genes. Gene expression levels were normalized using the following formula:

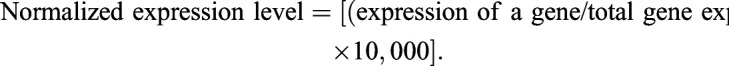


The values used to generate the feature and violin plots were calculated as log (normalized expression level+1). To mitigate the effects of cell cycle variation, the expression levels of cell cycle-related genes were regressed out from the analysis.

The average expression level and dispersion values were used to identify highly variable genes. Principal component analyses (PCAs) were performed on the detected genes for dimensional reduction. Forty PCAs were initially performed, and ten PCAs were used for the further analysis of cluster 7 cells. We used the shared nearest neighbor method to classify the cells into different clusters. To visualize the data in a two-dimensional space, we further reduced the dimension of the dataset using t-SNE. Clusters with <30 cells were eliminated from the analysis.

Marker genes in specific clusters were identified as those with log fold changes ≧0.25 and adjusted *P*-values <0.001 using the following formula (DEGs in Table S1): 

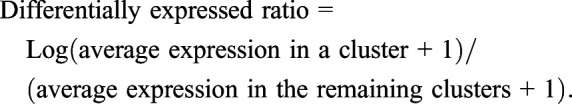
DEGs between *Mab21l1-*KO and wild type were identified using the following formula (DEGs in Table S2): 

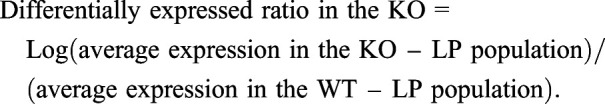


A likelihood-ratio test was performed to examine the significant differences in DEGs using Model-based Analysis of Single-cell Transcriptomics (MAST) (Finak et al., 2015). Adjusted *P*-values were calculated using the Bonferroni method. The software tools used in this study were Cell Ranger ver. 2.2, R ver. 3.5.0, Seurat ver. 2.3.4, Dplyr ver. 0.7.6 and MAST ver. 1.8.2.

## Supplementary Material

Supplementary information
