# Supplementary material for: MAB21L1 modulates gene expression and DNA metabolic processes in the lens placode
Source: Dis Model Mech. 2021 Dec 23;14(12):dmm049251. doi: 10.1242/dmm.049251 (PMC8713989; doi:10.1242/dmm.049251)
Supplement: Supplementary information [file dmm-14-049251-s1.pdf]

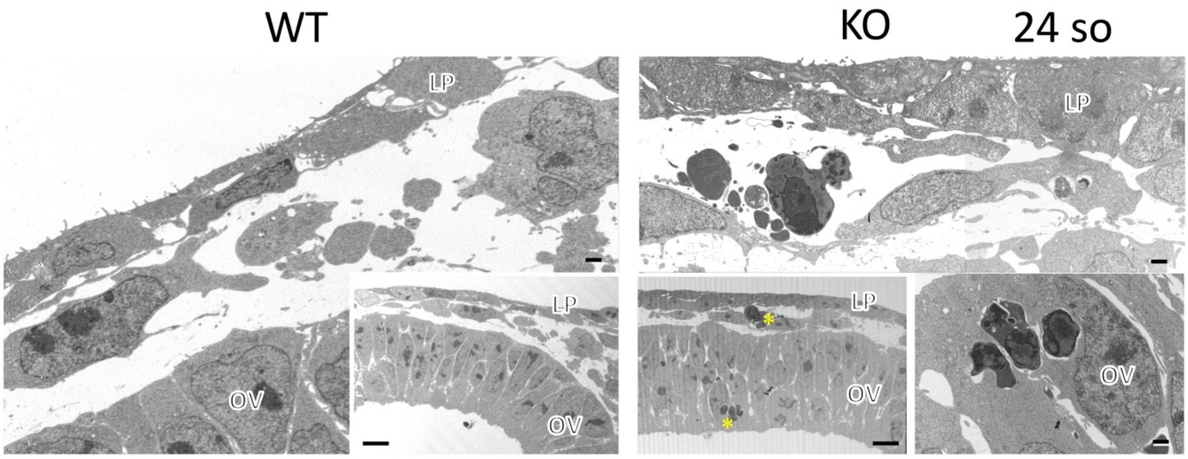

Fig. S1. Transmission electron microscopy (EM) reveals the ultrastructural phenotypes of *Mab21L1*-knockout (KO) lens placodes.

Transmission EM images of WT and KO lens ectodermal areas at the 24-somite stage (WT, n = 4; KO, n = 5). No ultrastructural defects were detected in the KO lens ectoderm, except for some apoptotic cell death in the lens field. Asterisk, cell debris; LP, lens placode; OV, optic vesicle. Scale bars: 1µm for main plates, 10 µm for lower-magnified images.

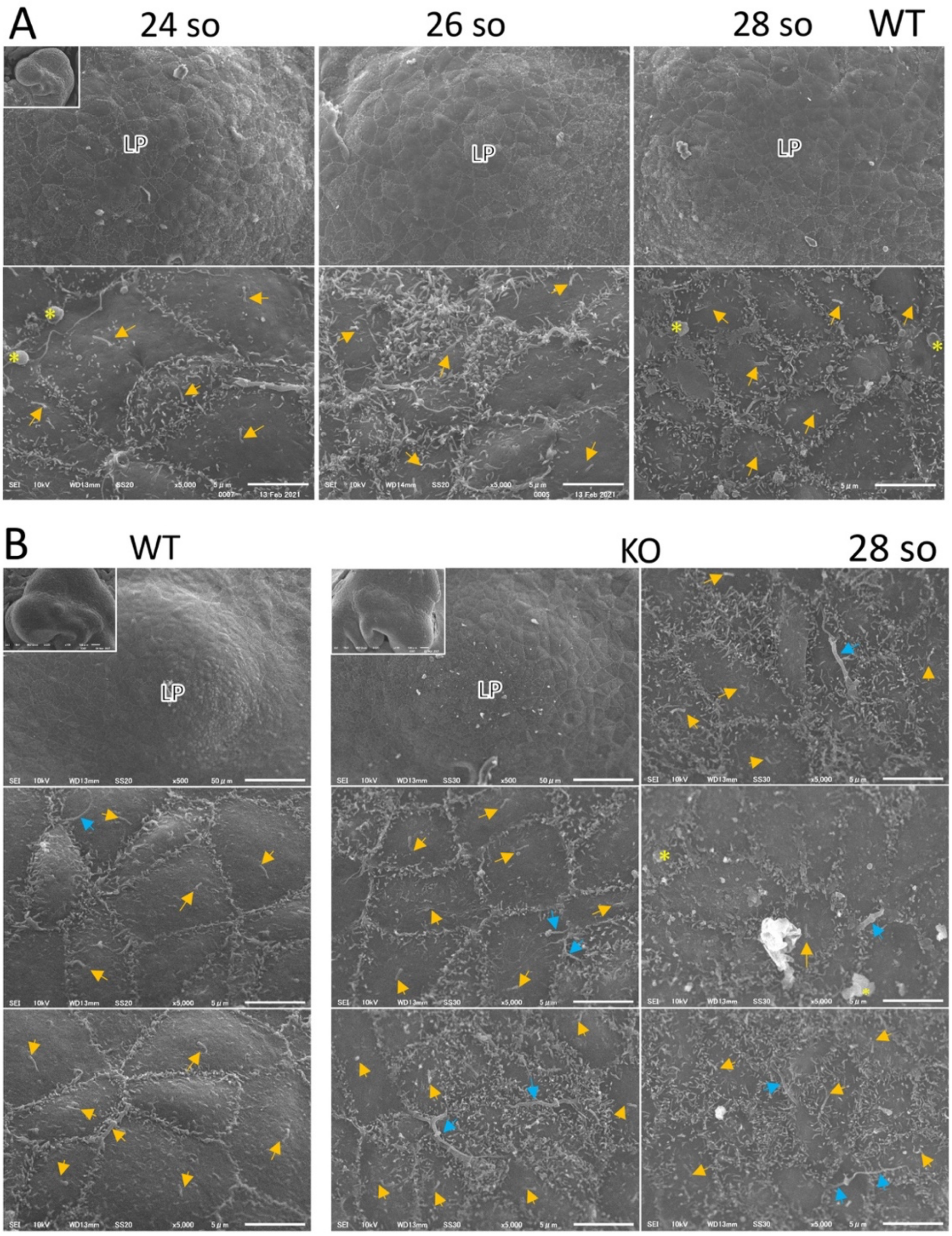

Fig. S2. Scanning electron microscopy (EM) reveals the ultrastructural phenotypes of *Mab21L1*-knockout (KO) lens placodes.

(A) Scanning EM images of WT lens ectodermal areas at the 24-, 26-, and 28-somite stages. the WT lens ectoderm tissues showed typical apical surface structures including one primary cilium (yellow arrows) and border microvilli (Figure 2C, upper panels). Also note some LP cells with numerous small protrusions/microvilli on their apical surface especially at 24- and 26-somite stages (i.e., prior to placode formation). (B) Scanning EM images of WT and KO lens ectodermal areas at the 28-somite stage (WT, n = 3; KO, n= 4). The WT and KO lens ectoderm tissues showed typical apical surface structures including one primary cilium (yellow arrows) and border microvilli. Besides the long cell protrusions (blue arrows), numerous small protrusions/microvilli are found in some KO cells even at 28-somite stage, showing similar to those of WT cells at 24-26-somite stages in A. Asterisk, cell debris; LP, lens placode. Scale bars: 5 μm or 50 μm as indicated.

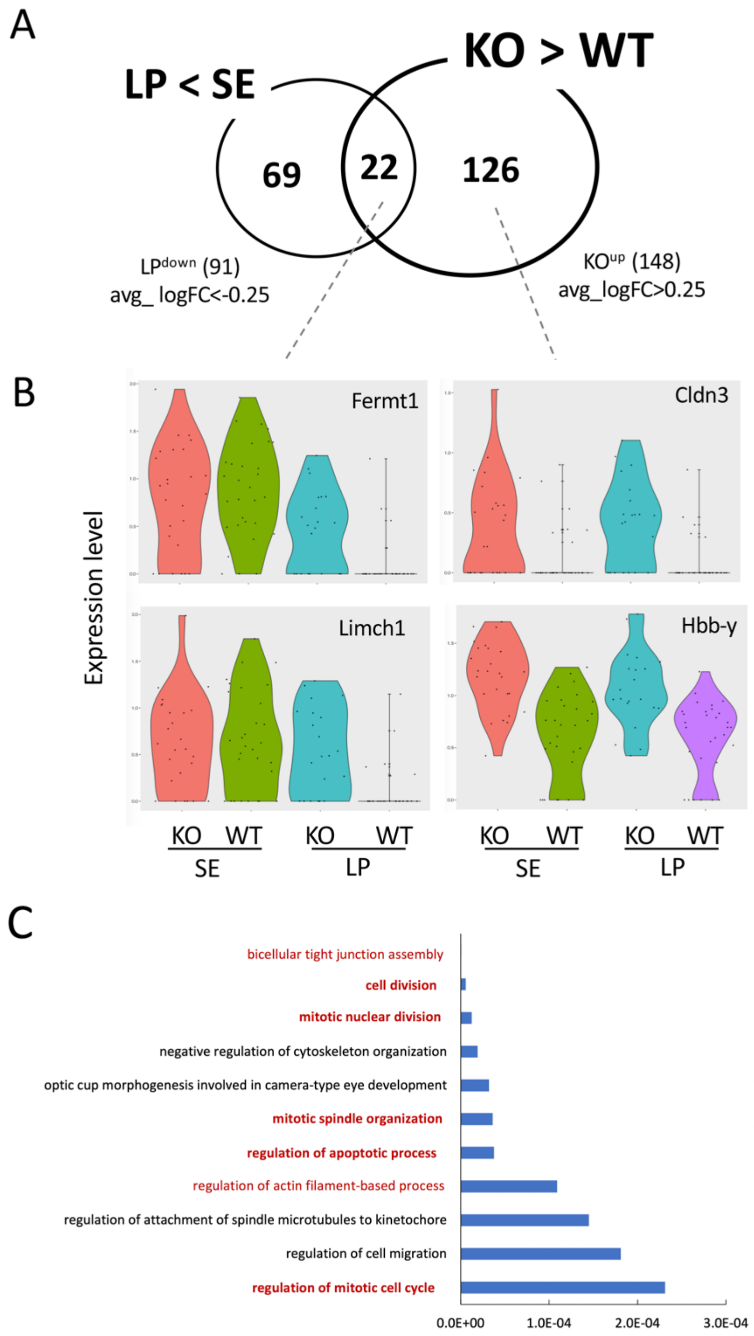

Fig. S3. The identification of 22 upregulated surface ectoderm (SE) genes in *Mab21l1*-null (KO) lens placodes (LPs).

(A) Venn diagram showing the 91 genes upregulated in SE and the 148 genes upregulated in KO (KO^{up}); 22 SE-specific genes are upregulated in both the KO and LP cell groups ($KO\text{-}LP^{up}$). (B) Violin plots showing the expression levels of *Fermt1*, *Limch1*, *Cldn2*, and *Hbb-y*, as representative genes upregulated in the KO cluster compared to the WT cluster. (C) 11 gene ontology (GO) terms associated with the 148 KO^{up} genes, with lens/eye-related GO terms omitted. GO terms mentioned in the Results and Discussion sections are highlighted in bold font.

Disease Models & Mechanisms • Supplementary information

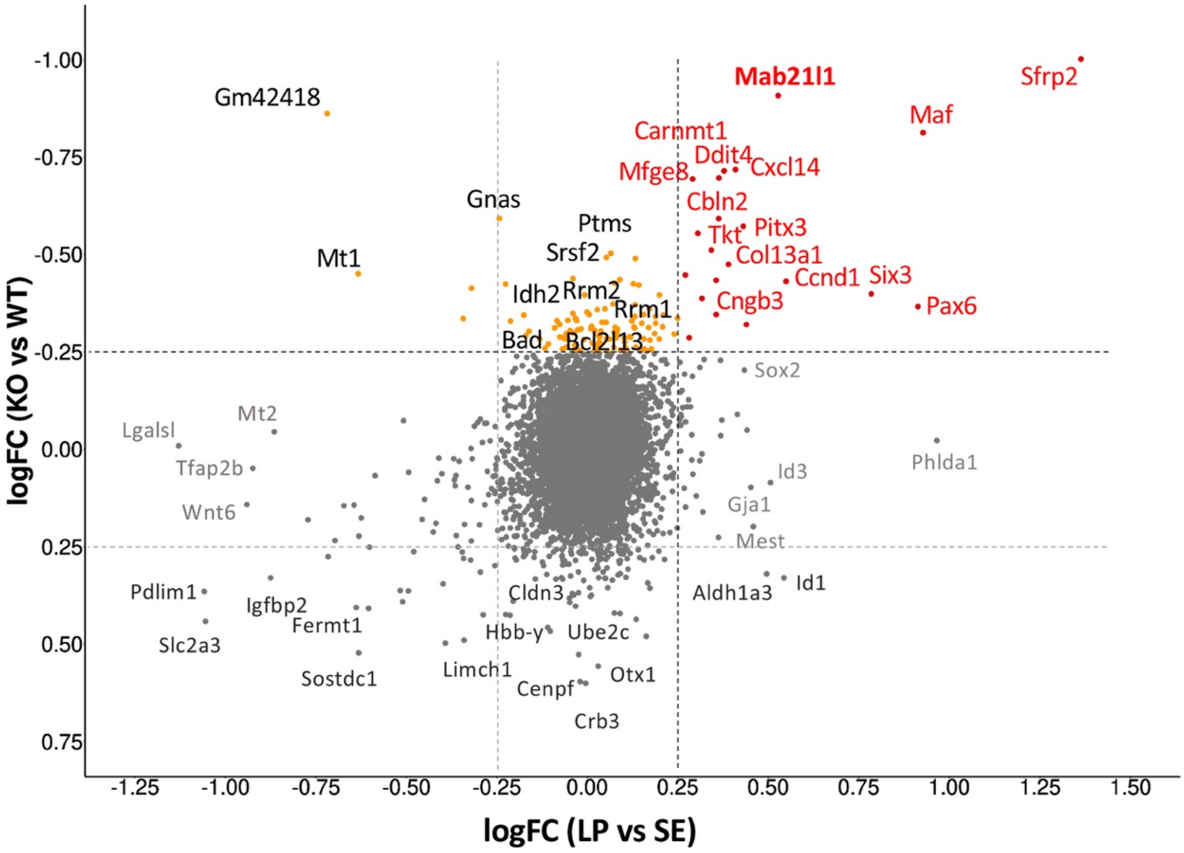

Fig. S4. Scatter plot showing the LogFC relative gene expression of *Mab21l1*-null (KO)/ wild-type (WT) cells against the LogFC relative gene expression of lens placode (LP) cells vs. surrounding surface ectoderm (SE) cells. Red dots represent the 21 KO-LP^{down} genes shared by the KO and LP groups, as shown in the Venn diagram in Figure 4C. Orange dots represent the rest of the KO^{down} genes (110 genes). The names of representative genes are indicated near the corresponing dot. Dot lines: logFC = -0.25 and logFC = 0.25.

Disease Models & Mechanisms • Supplementary information

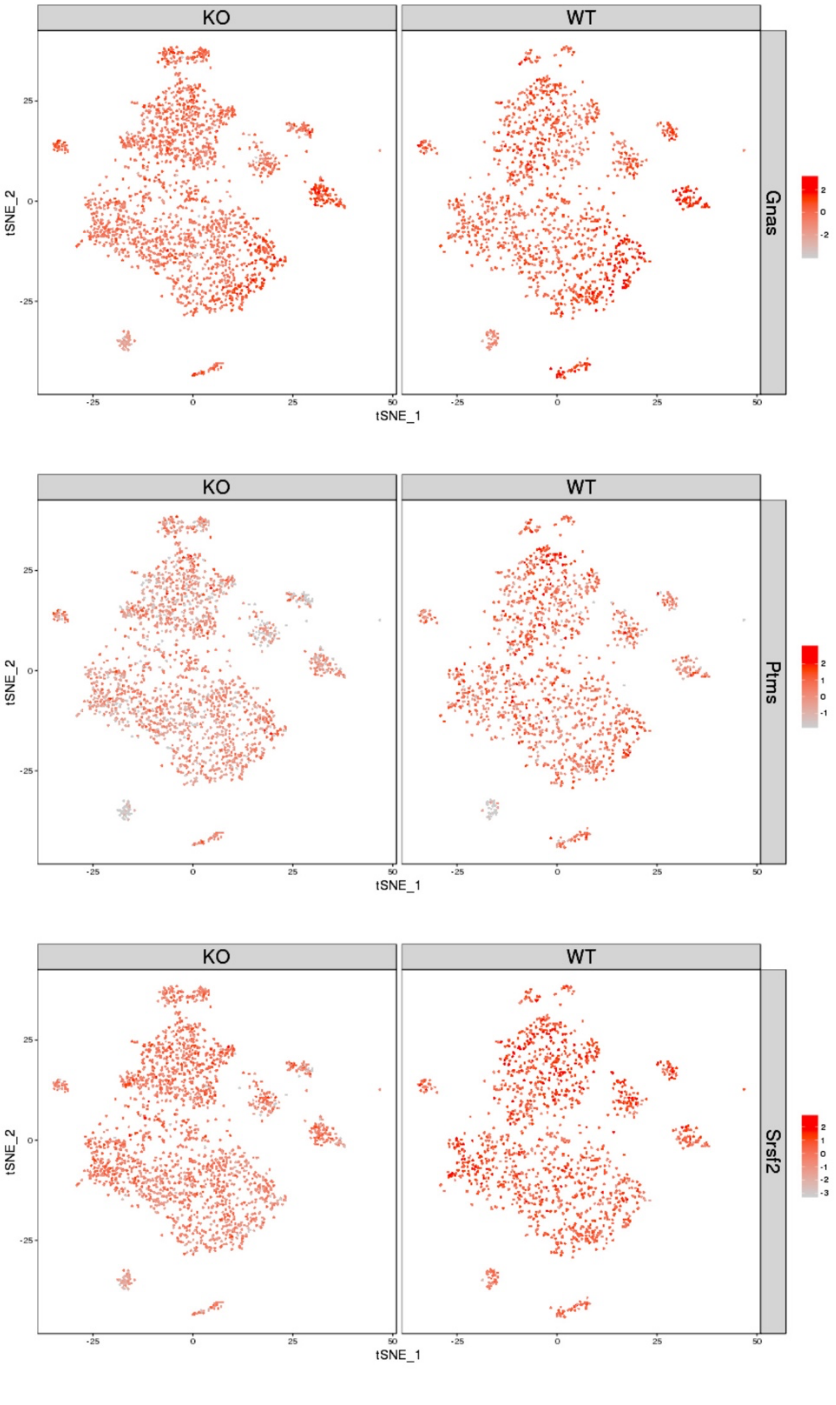

Fig. S5. T-distributed stochastic neighbor embedding (t-SNE) feature plot showing the ubiquitous expression of *Gnas*, *Ptms* and *Srsf2* in KO and WT embryo cells.

Disease Models & Mechanisms • Supplementary information

Table S1.

Click here to download Table S1

Table S2.

Click here to download Table S2

Table S3.

Click here to download Table S3

Table S4. The 21 lens placode (LP) genes that are down-regulated in *Mab21l1*-null ectoderm (KO-LP^{down}) [1).]

	KO < WT		LP > SE	
gene_symbol [2)]	avg_logFC	p_val	avg_logFC	p_val
**Sfrp2**	-1.0012466	4.72E-06	1.36341068	1.54E-12
**Mab21l1**	-0.9075676	2.02E-14	0.526888	1.30E-06
**Maf**	-0.8127795	1.53E-05	0.92739914	1.83E-09
Cxcl14	-0.718169	5.19E-06	0.40924747	0.00089045
Ddit4	-0.7149604	7.48E-05	0.37749042	0.00404634
Carnmt1	-0.6967923	1.53E-05	0.36325424	0.00493539
Mfge8	-0.6940889	0.00092808	0.29075347	0.09995295
Cbln2	-0.5916955	1.99E-06	0.36266753	0.00038213
**Pitx3**	-0.5719038	5.29E-05	0.43078029	9.51E-08
Tkt	-0.5537238	0.00032429	0.30533229	0.00547852
Col13a1	-0.5107722	0.00071387	0.3426715	0.00017564
Ldha	-0.4745407	0.00959348	0.38952393	0.00266779
Has2	-0.4470154	0.00090494	0.27060805	0.00328181
Cngb3	-0.433732	0.00430548	0.35583483	3.02E-06
Ccnd1	-0.430996	0.02077395	0.54904706	0.00013579
**Six3**	-0.398843	0.03504901	0.78430365	1.30E-11
Stra6	-0.387143	0.01847258	0.31666258	0.001286
**Pax6**	-0.366096	0.01995318	0.91327293	1.83E-12
Dkk3	-0.345693	0.06050467	0.35587081	0.00123721
Flrt2	-0.320127	0.07185614	0.43922041	2.16E-05
Vcan	-0.286523	0.10106065	0.28149998	0.00264515

[1)] KO-LP^{down} corresponds to the shared region of Venn diagram in Fig. 4C.

[2)] The gene list is ordered by avg_logFC in KO < WT. Gene symbols with bold letters indicate the key genes crucial for lens placode formation.

Disease Models & Mechanisms • Supplementary information
